# Effects of Dietary Resveratrol Supplementation on Fermentation Characteristics, Microbial Diversity, and Community Composition of Feces in Hu Sheep

**DOI:** 10.3390/ani15172494

**Published:** 2025-08-25

**Authors:** Dan Luo, Lin Li, Chengjing Cui, Kehui Ouyang, Mingren Qu, Qinghua Qiu

**Affiliations:** Jiangxi Province Key Laboratory of Animal Nutrition and Feed, College of Animal Science and Technology, Jiangxi Agricultural University, Nanchang 330045, China

**Keywords:** dietary intervention, metabolic pathway, microbial biomarker, ruminant production, sheep

## Abstract

This study aimed to investigate the effects of dietary resveratrol supplementation on fermentation characteristics, microbial diversity, and community composition of feces from Hu sheep. A total of 20 three-month-old Hu sheep with similar body weights (20.62 ± 0.51 kg) were randomly divided into the control group (fed a basal diet, CON) and the treatment group (fed a basal diet supplemented with resveratrol at 100 mg/kg of feed, RES), with 10 sheep in each group, and lasted for 75 days. Feces were collected from each sheep at twenty-four time points for fecal fermentation characteristics determination and microbial analysis. The results showed that the pH value was higher in the RES group than in the CON group (*p* < 0.05), while the concentration of ammonia nitrogen was lower, showing a 10.6% reduction compared to the CON group (*p* = 0.013). The richness, Shannon index, and inverse Simpson index of fecal microbiota were higher in the CON group than in the RES group (*p* < 0.05). The relative abundances of Planctomycetota, *Bacteroides*, *Alistipes*, and *NK4A214 group* were higher in the CON group than in the RES group (*p* < 0.05). Additionally, the relative abundance of the glycan biosynthesis and metabolism pathway was higher in the CON group (*p* < 0.05). The relative abundance of *Prevotella* was lower in the CON group than in the RES group (*p* < 0.05). Principal co-ordinate analysis (PCoA) revealed no overlap between the two groups, and analysis of similarities (ANOSIM) showed significant differences between the CON and RES groups (R = 0.4560, *p* = 0.012). Linear discriminant analysis effect size (LEfSe) analysis identified 27 microbial biomarkers, with the RES group having more beneficial bacteria and the CON group having more potentially harmful bacteria. The study demonstrated that dietary resveratrol supplementation reduced the concentration of ammonia nitrogen in feces, decreased microbial diversity, and increased the abundance of beneficial bacteria. The findings of this research provide a post-digestion perspective for evaluating the application of resveratrol in ruminant production.

## Simple Summary

Resveratrol, a polyphenolic compound, is renowned for its antioxidant, anti-inflammatory, and anticancer properties. Its incorporation into the diets of ruminants has been well-documented to enhance growth performance and overall animal health. Despite these benefits, the potential post-digestion impact of dietary resveratrol supplementation remains largely unexplored in the literature. In this study, the effects of dietary resveratrol supplementation on fermentation characteristics, microbial diversity, and community composition of feces in Hu sheep were investigated. The results revealed that resveratrol supplementation significantly reduced fecal ammonia nitrogen, decreased microbial diversity, and increased abundances of beneficial bacteria. These findings not only highlight the positive effects of resveratrol on beneficial bacteria, but also provide valuable insights into its post-digestion implications. This study offers an understanding of implications in adding dietary reseveratrol on reducing fecal ammonia nitrogen.

## 1. Introduction

Ruminants are unique in their ability to digest fibrous plant materials through their specialized multi-chambered stomach, particularly the rumen. The rumen houses a diverse community of microorganisms that ferment and break down cellulose. This fermentation process produces volatile fatty acids (VFA), which serve as a primary energy source for ruminants [1]. However, optimizing ruminal fermentation and overall digestive efficiency remains a challenge. Previous studies have shown that resveratrol, a plant secondary metabolite, can enhance ruminal fermentation by increasing the production of VFA and improving the digestibility of nutrients [2,3,4]. Additionally, resveratrol has been found to boost the immune system of ruminants by increasing immunoglobulin levels, which can reduce disease incidence and support long-term growth [5]. These findings suggest that resveratrol holds promise as a feed additive to improve the production performance of ruminants by leveraging their unique digestive system.

Ruminant fecal characteristics have the potential to serve as indirect indicators of nutrient utilization efficiency, as they may reflect the byproducts of nutrient absorption and digestion within the lower gastrointestinal tract [6]. The inclusion of resveratrol in the diet offers a novel approach to modulate gut fermentation characteristics and microbial composition, potentially fostering beneficial bacterial populations and enhancing overall gut health [3]. By improving nutrient digestion and absorption, resveratrol may alter the fermentation characteristics and microbial composition of ruminant feces. This alteration occurs because nutrient utilization efficiency directly influences the substrates available for microbial fermentation in the lower digestive tract [6]. Additionally, resveratrol’s modulation of the gut microbiota could further influence the fermentation process and the microbial community structure in feces. Thus, it is plausible that resveratrol’s impact on nutrient utilization efficiency is also reflected in changes to the fermentation characteristics and microbial profiles of ruminant feces. However, there is limited information on how resveratrol supplementation specifically affects these fecal fermentation characteristics and microbial profiles.

Animal waste, particularly from livestock, is a significant contributor to environmental pollution. The emission of organic acids and ammonia from this waste can lead to water contamination and soil degradation [7]. Such pollutants not only impair water quality, but also contribute to the eutrophication of aquatic ecosystems [8]. This process can lead to oxygen depletion, which, in turn, causes the death of aquatic organisms [7]. When ammonia nitrogen percolates into groundwater or seeps into surface water, it has the potential to contaminate drinking water sources, posing a significant threat to human health [9]. The direct application of untreated animal feces to soil can lead to soil acidification and compaction [10]. The volatilization of ammonia nitrogen from animal feces into ammonia gas significantly contributes to air pollution. Moreover, ammonia nitrogen and the ammonia gas generated in animal pens can induce diseases and adversely affect the growth and development of animals. Ruminant fecal ammonia nitrogen emissions account for as much as 25% of agricultural non-point source pollution [11,12]. Consequently, it is imperative to consider the influence of resveratrol on the fermentation characteristics and microbial composition of ruminant feces when evaluating its impact on enhancing nutrient utilization efficiency. These factors have a direct bearing on the environmental footprint of animal agriculture, which necessitates a comprehensive assessment of the ecological implications associated with the use of resveratrol as a feed additive. Therefore, it is crucial to further investigate the impact of resveratrol supplementation on fecal fermentation characteristics and the microbial community. This exploration will be instrumental in undertaking a more comprehensive assessment of resveratrol’s practical applications within ruminant production.

To this end, a 75-day feeding trial was conducted to investigate the effects of dietary resveratrol supplementation on fermentation characteristics, microbial diversity, and community composition of feces in Hu sheep. It was hypothesized that the inclusion of resveratrol in the diet would affect ammonia nitrogen emission, microbial diversity, and community composition. The findings can provide a post-digestion perspective for evaluating the application of resveratrol in ruminant production.

## 2. Materials and Methods

### 2.1. Animal Care

This research was carried out in full compliance with the guidelines for animal care and welfare, and received approval from the Institutional Animal Care and Use Committee (IACUC) of Jiangxi Agricultural University, with the protocol number JXAULL-20240340.

### 2.2. Experimental Design

Twenty healthy female Hu sheep, with similar body weights (20.62 ± 0.51 kg) and ages (90.00 ± 0.30 days), were randomly assigned to two groups, each comprising 10 sheep. One group was given a basal diet, whereas the other group was fed the same basal diet supplemented with resveratrol at 100 mg/kg of feed. The purity of resveratrol was 98.5% and was provided by Herbchem Biotech Co., Ltd. (Xi’an, China). The feed composition and nutritional content of the basal diet is presented in Table 1. The experimental animals were acclimated to the same diet for 15 days prior to the experiment, and were then fed according to the assigned treatments for 60 days. Feed was provided ad libitum, and clean and abundant drinking water was available at all times. The daily feed allowance was adjusted to ensure that the residual feed on the following day accounted for 5% to 10% of the total feed offered. It is worth noting that all animals remained healthy throughout the trial.

### 2.3. Sample Collection

Feed samples from three randomly selected days each week were collected for chemical composition analysis. For five consecutive days prior to the trial end, fecal samples were collected from each sheep using rectal spot sampling [13,14]. Specifically, over the first four days, fecal samples were collected every six hours (4 times/day × 4 days = 16 samples). The time point were scheduled as 0:00, 6:00, 12:00, and 18:00 on days 1 and 3; and 3:00, 9:00, 15:00, and 21:00 on days 2 and 4. On the fifth day, fecal samples were collected every three hours (8 times/day × 1 day = 8 samples). The 24 fecal samples were then thoroughly mixed to create a composite sample representing the individual animal, which was stored in a −80 °C freezer.

### 2.4. Feed Analysis and Fecal Fermentation Characteristic Determination

The concentrations of crude protein (CP; method 2001.11), ether extract (EE; method 945.16), calcium (method 935.14), and phosphorus (method 942.23) in the feed were assessed using AOAC International methods [15]. Neutral detergent fiber (NDF) and acid detergent fiber (ADF) were analyzed according to the procedures outlined by Van Soest et al. [16], with the inclusion of thermostable *α*-amylase. Following the method of Sato and Nakajima [17], 5 g of feces was dissolved in 20 mL of sterile water and homogenized using a homogenizer. The pH value of the feces was immediately measured using a portable pH meter (testo 206, testo AG, Schwarzwald, Germany). First, the homogenized mixture was centrifuged at 6000× *g* for 30 min to obtain the supernatant, which was then centrifuged again at 20,000× *g* for 20 min. The resulting supernatant was used as the sample for the subsequent determination of ammonia nitrogen (NH_3_-N) and VFA. As reported by Broderick and Kang [18], NH_3_-N was determined by the phenol–hypochlorite colorimetric method. The determination of VFA was performed using an Agilent 8860 gas chromatograph (Agilent Technologies, Inc., Santa Clara, CA, USA) equipped with a 30 m HP INNOWAX capillary column (19091N-2131, 0.32 mm internal diameter × 0.50 μm film thickness) and a flame ionization detector (FID-2019, Agilent Technologies, Inc., Santa Clara, CA, USA). Nitrogen served as the carrier gas, with a flow rate of 2.5 mL/min. The injection volume was 1.0 μL, and the injector temperature was set at 235 °C. The oven temperature was programmed as follows: it was held at 100 °C for 30 s, then ramped to 180 °C over 10 min and maintained for 2 min, followed by a linear increase to 260 °C at a rate of 10 °C/min. The split ratio was maintained at 40:1. Analytical standards for quantification were obtained from Sigma-Aldrich (Merck KGaA, Darmstadt, Germany). The identity of each VFA (acetate, propionate, isobutyrate, butyrate, isovalerate, and valerate) was verified based on its relative retention time, and the concentration of each VFA was quantified using the external standard method.

### 2.5. Fecal DNA Extraction, Sequencing, and Analysis

Fecal DNA extraction was performed using the QIAGEN PowerFecal Pro DNA Kit (QIAGEN, Hilden, Germany). The concentration and purity of the extracted DNA were determined using a Qubit 3.0 fluorometer (Life Technologies, Carlsbad, CA, USA) and a NanoDrop ND-2000 spectrophotometer (Thermo Fisher Scientific, Waltham, MA, USA), respectively. DNA integrity was evaluated using 1% agarose gel electrophoresis. The full-length bacterial 16S rRNA gene was amplified by PCR using the forward primer 27F (5′-AGRGTTYGATYMTGGCTCAG-3′) and the reverse primer 1492R (5′-RGYTACCTTGTTACGACTT-3′). Sample-specific 16bp barcodes were incorporated into the primers for multiplex sequencing. The PCR reaction mixture included 5 μL KAPA HiFi buffer (5×), 0.5 μL KAPA HiFi HotStart DNA polymerase (1 U/μL), 1 μL (10 mM) dNTPs, 1 μL (10 μM) of each forward and reverse primer, 2 μL DNA template, and 15.5 μL ddH_2_O. The PCR protocol involved an initial denaturation at 95 °C for 5 min, followed by 30 cycles consisting of denaturation at 95 °C for 30 s, annealing at 57 °C for 30 s, and extension at 72 °C for 60 s, with a final extension at 72 °C for 5 min. The PCR amplicons were purified using AMPure XP beads (Beckman Coulter, Indianapolis, IN, USA) and quantified with the Quant-iT dsDNA HS Assay Kit (Invitrogen, Carlsbad, CA, USA). The library was prepared with the SMRTbell Express Template Prep Kit 2.0-SPv4. After passing quality control checks, the library was sent to BAXBio Technology Co., Ltd. (Beijing, China) for sequencing on the PacBio Sequel II platform. It is important to highlight that, due to budget constraints, the 10 fecal samples in each group were randomly combined in pairs to create 5 new composite samples per group. Consequently, a total of 10 composite samples were ultimately utilized for DNA extraction and sequencing analysis in this study. The raw sequencing data of these 10 samples were deposited in the NCBI Sequence Read Archive (SRA) database, and the assigned accession number is PRJNA1274161.

The raw sequencing data were initially processed using the SMRT Link software (version 11.0) to split the samples based on the barcode, to generate high-fidelity (HiFi) reads. Subsequently, the DADA2 denoising algorithm was employed to produce amplicon sequence variants (ASVs), which served as the foundation for the taxonomic identification of species. For the annotation process, the SILVA 138 database was used in conjunction with the classify-sklearn algorithm from QIIME 2. To quantify the species diversity within the habitat, alpha diversity metrics were calculated, including richness, Chao1, ACE, Shannon index, Simpson index, and inverse Simpson. These metrics were derived using the “qiime diversity alpha” workflow in QIIME 2. Principal co-ordinate analysis (PCoA) was used to elucidate the disparities in microbial community composition between the CON and RES groups. Specifically, PCoA plots were constructed based on both Bray–Curtis and Jaccard distances to provide a comprehensive comparison. Furthermore, the analysis of similarities (ANOSIM) was conducted to statistically evaluate the differences in microbial community structure between the CON and RES groups. It used the Bray–Curtis dissimilarity matrix to compare the distances within and between these two groups. Linear discriminant analysis effect size (LEfSe) was used to pinpoint microbial biomarkers that distinguish the CON and RES groups across various taxonomic ranks. Initially, the non-parametric Kruskal–Wallis rank-sum test was conducted to identify significant disparities in species abundance between the CON and RES groups. This was followed by the Wilcoxon rank-sum test to confirm the consistency of these differences. Lastly, linear discriminant analysis (LDA) was employed to assess the effect size of these differences on group discrimination, with the LDA score threshold set at 3. Phylogenetic Investigation of Communities by Reconstruction of Unobserved States 2 (PICRUSt2, version 2.6.0) was employed to predict the metagenomic contributions of the microbial communities in the CON and RES groups, thereby elucidating their functional profiles and revealing the intrinsic differences between the two groups.

### 2.6. Statistical Analysis

All data were initially tested for normality using the Shapiro–Wilk test. For data that followed a normal distribution (*p* > 0.05), a *t*-test was used to analyze differences between the CON and RES groups. For indicators that did not follow a normal distribution (*p* < 0.05), the Mann–Whitney U test was used to assess differences between the two groups. All analyses were conducted using SPSS software (version 20, IBM, Chicago, IL, USA), with a significance level set at *p* < 0.05.

## 3. Results

### 3.1. Fecal Fermentation Characteristics

The effect of resveratrol supplementation on the fecal fermentation characteristics of Hu sheep is listed in Table 2. The fecal pH value in the RES group was higher than that of the CON group, while the concentration of NH_3_-N was lower compared with that of the CON group (*p* < 0.05). The inclusion of resveratrol had no effect on the concentrations of either individual VFA or total VFA, nor did it affect the proportion of individual VFA (*p* > 0.05).

### 3.2. Fecal Bacterial Alpha-Diversity

The effect of resveratrol supplementation on the fecal bacterial alpha-diversity indices of Hu sheep is shown in Table 3. The RES group had lower richness, Shannon index, and inverse Simpson index compared with the CON group (*p* < 0.05).

### 3.3. Fecal Bacterial Community Composition

The effects of resveratrol supplementation on fecal bacterial community composition at the levels of phylum and genus are presented in Table 4 and Table 5, respectively. Firmicutes and Bacteroidota are the two phyla with the highest relative abundance, with relative abundances of 58.8% and 31.14%, respectively. The relative abundance of Planctomycetota was higher in the CON group than in the RES group (*p* < 0.05). Resveratrol supplementation had no significant effect on other phyla with a relative abundance of more than 0.1% (*p* > 0.05). At the genus level, *Christensenellaceae R-7 group* and *Rikenellaceae RC9 gut group* are the two dominant microbes, accounting for 12.85% and 9.06%, respectively. The relative abundance of *Prevotella* in the RES group was higher than that in the CON group, while it was lower in *Bacteroides*, *Alistipes*, and *NK4A214 group* than in the CON group (*p* < 0.05).

### 3.4. Fecal Bacterial Beta-Diversity

**Figure 1:**
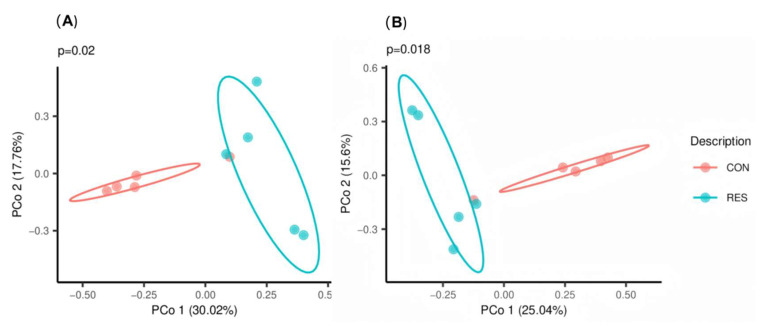
Effect of dietary resveratrol supplementation on the principal co-ordinate analysis (PCoA) of the fecal bacteria in Hu sheep based on (**A**) Bray–Curtis and (**B**) Jaccard distances. CON, the control group which was given a basal diet; RES, the treatment group which was fed the same basal diet supplemented with resveratrol at 100 mg/kg of feed.

The PCoA results based on the Bray–Curtis and Jaccard dissimilarity indices showed no overlap between the CON and RES groups (Figure 1). ANOSIM also detected significant differences between the two groups (R = 0.4560, *p* = 0.012).

### 3.5. Microbial Biomarker

**Figure 2:**
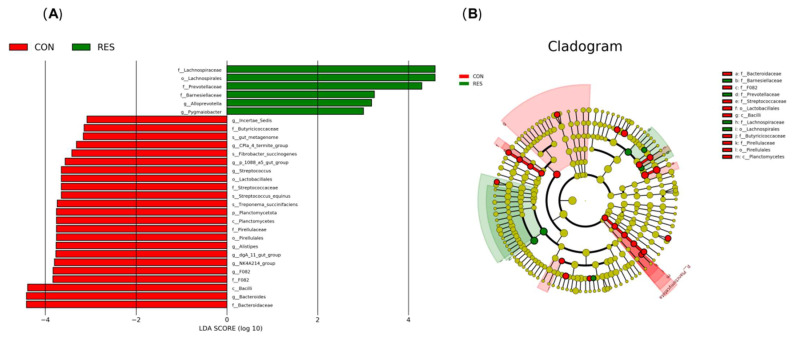
Effect of dietary resveratrol supplementation on the discriminative bacterial communities at different taxonomic levels in the feces of Hu sheep. (**A**) Linear discriminant analysis (LDA); (**B**) Cladogram. CON, the control group which was given a basal diet; RES, the treatment group which was fed the same basal diet supplemented with resveratrol at 100 mg/kg of feed.

The LDA effect size analysis (Figure 2) identified a total of 27 differential species between the CON and RES groups, with 21 species in the CON group and 6 species in the RES group. The marker microorganisms in the RES group included f__Lachnospiraceae, o__Lachnospirales, f__Prevotellaceae, f__Barnesiellaceae, g__*Alloprevotella*, and g__*Pygmaiobacter*. The marker microorganisms in the CON group included f__Butyricicoccaceae, g__*CPla 4 termite group*, s__*Fibrobacter succinogenes*, g__*p 1088 a5 gut group*, g__*Streptococcus*, o__Lactobacillales, f__Streptococcaceae, s__*Streptococcus equinus*, s__*Treponema succinifaciens*, p__Planctomycetota, c__Planctomycetes, f__Pirellulaceae, o__Pirellulales, g__*Alistipes*, g__*dgA 11 gut group*, g__*NK4A214 group*, g__*F082*, f__F082, c__Bacilli, g__*Bacteroides*, and f__Bacteroidaceae.

### 3.6. Predicted Metabolic Pathway

The effect of resveratrol supplementation on the relative abundance of the predicted metabolic pathways in the fecal bacterial community is shown in Table 6. The relative abundance of glycan biosynthesis and metabolism was lower in the RES group than in the CON group (*p* < 0.05), while no significant differences were observed between the RES and CON groups for other metabolic pathways with relative abundances greater than 1% (*p* > 0.05).

## 4. Discussion

Ammonia nitrogen in animal feces exerts a wide range of detrimental impacts on both the environment and animal health. Therefore, reducing ammonia nitrogen levels in animal feces is highly desirable in animal husbandry. In this study, the dietary addition of resveratrol was found to reduce the concentration of NH_3_-N in feces. This reduction may be attributed to more complete digestion of nutrients, particularly proteins, in the foregut. As previous studies have shown that resveratrol can enhance nutrient digestibility [4]. However, future studies require ileal cannulation sampling to validate amino acid digestibility. In this study, the reduction in ammonia nitrogen was not accompanied by a synchronous decrease in branched-chain VFA. The possible reasons for this could be that the products of protein degradation also include branched-chain amino acids, and it is also possible that higher microbial nitrogen utilization was converted into microbial protein and other metabolic products. A more detailed analysis of nitrogen-containing metabolites could provide a more comprehensive understanding of the mechanism behind the reduction in ammonia nitrogen. Correspondingly, branched-chain VFA serve as a crucial source of carbon and energy for cellulolytic and hemicellulolytic rumen bacteria [19]. The higher numerical concentration of isobutyrate and the proportion of branched-chain VFA in the RES group can account for the higher relative abundance of *Prevotella* in that group, given that *Prevotella* is recognized as a fiber-degrading genus [20]. Besides, *Prevotella* also participate in protein degradation and can utilize branched-chain amino acids. Therefore, the enrichment of *Prevotella* does not necessarily lead to changes in VFA, this phenomenon can be further verified by the fact that there were no significant differences in the predicted abundance of acetate, propionate, and butyrate metabolism between the CON and RES groups. The fecal pH plays a dual role in soil health, as it regulates the soil’s acidity or alkalinity and influences the availability of soil nutrients. Within the range of 6.5 to 7.5, the fecal pH exerts the most favorable effect on plant growth [21]. In this study, the fecal pH remained within the optimal range. Upon the addition of resveratrol, the fecal pH increased, potentially due to the numerically lower concentration of VFA in the feces.

Richness directly indicates the quantity of microbial species present. In contrast, the Shannon index not only accounts for species richness but also incorporates species evenness. Similarly, the inverse Simpson index factors in both species richness and evenness. Moreover, it is more responsive to changes in dominant species, enabling it to more precisely capture the influence of a few dominant species on community diversity [22]. In this study, it was observed that the addition of resveratrol led to a reduction in all three metrics within the fecal microbiota, which suggests that resveratrol supplementation decreases alpha diversity. One plausible explanation is that resveratrol has been shown to increase the abundance of beneficial bacteria while reducing the levels of potentially harmful bacteria [2]. Moreover, the functional specialization of the microbiota may help the host better adapt to environmental changes and improve the host’s health status. Therefore, this shift can result in a more uniform microbial community structure and optimized function, thereby enhancing the overall functional efficiency of the microbial community.

Planctomycetota is the sole known bacterial phylum with anammox capabilities. Anammox is a significant nitrogen cycling process that converts ammonium nitrogen (NH_4_^+^) and nitrite nitrogen (NO_2_^−^) into nitrogen gas (N_2_), without producing the greenhouse gas nitrous oxide (N_2_O) [23]. In this study, it was found that the relative abundance of Planctomycetota was higher in the CON group, indicating that more nitrogen in this group requires further decomposition and utilization, which corresponds to the higher concentration of NH_3_-N in this group. *Bacteroides* is the largest genus within the phylum Bacteroidetes and is known for its ability to break down complex polysaccharides into short-chain fatty acids (SCFA), which serve as a primary energy source for animal intestinal epithelial cells [24]. However, *Bacteroides fragilis*, a member of this genus, can become pathogenic and cause infections when the immune system is compromised or the intestinal barrier function is disrupted [25]. Similarly, *Alistipes*, another genus within the phylum Bacteroidetes, is involved in nitrogen metabolism and can break down complex carbohydrates to produce succinate, acetate, and propionate. Despite these beneficial metabolic functions, *Alistipes* has been shown to exhibit pro-inflammatory effects in diseases such as colorectal cancer, depression, and Parkinson’s disease [26]. In this study, it was found that the relative abundances of *Bacteroides* and *Alistipes* was higher in the CON group. This increase may be attributed to the higher levels of undigested nutrients in this group, which support the growth of these bacteria. Additionally, the presence of more pathogenic bacteria in the CON group may lead to poorer health conditions. This finding is supported by the observation of lower serum antioxidant capacity in this group, which may indicate a higher inflammatory burden. *NK4A214 group*, belonging to the phylum Firmicutes and the family Oscillospiraceae, is an uncultured bacterial group that is extensively distributed within the rumen microbial community [27]. Previous research revealed that its abundance surges significantly in dairy goats suffering from subacute ruminal acidosis, hinting at its potential connection with the onset of the disease [28]. Moreover, its substantial correlation with methanogenic archaea in the rumen suggests that it might be a key player in the ruminal methane production process [29]. The relatively lower abundance of *NK4A214 group* in the RES group implies that the incorporation of resveratrol could boost immune capacity and energy utilization efficiency. This is supported by the fact that methane production represents a significant energy loss in ruminants [30]. *Prevotella* is capable of degrading complex carbohydrates and cellulose, and its production of SCFA exerts beneficial effects on gut health. Enriching *Prevotella* in the gut not only enhances feed conversion efficiency in animals but also mitigates methane emissions by fixing hydrogen through the fermentation of sugars or lactate [31]. Moreover, *Prevotella copri* has been found to bolster the body’s immune system and maintain health by modulating systemic immune responses and suppressing diseases [32]. In this study, the higher abundance of *Prevotella* in the RES group was associated with improved nutrient utilization and enhanced immune capacity following resveratrol supplementation. This suggests that resveratrol may promote the growth of beneficial bacteria, thereby contributing to better nutrient absorption and immune function.

LEfSe analysis provides a comprehensive approach to analyzing microbiome data across multiple taxonomic levels, and offers high sensitivity in detecting abundance variations. In addition to the differential microbes and their associated species discussed earlier, LEfSe analysis revealed 16 additional microbial biomarkers. Bacteria from the Lachnospiraceae family are capable of degrading complex, hard-to-digest materials such as cellulose and hemicellulose in feed, leading to the production of organic acids and VFA [33]. These bacteria are well recognized as primary producers of SCFA and are also known to generate immune-modulating metabolites and antigens that contribute to the regulation of the host’s immune system [33]. Numerous studies have demonstrated a positive correlation between the abundance of Lachnospiraceae and improved feed utilization efficiency, as well as a reduction in methane production [33,34]. In this study, higher abundances of both Lachnospiraceae and Lachnospirales were observed in the RES group, which were closely associated with enhanced feed efficiency and overall health status within this group. The balance and diversity of the Barnesiellaceae gut microbiota are intricately linked to health, with studies revealing significant enrichment in the ceca of high-weight broilers [35]. *Alloprevotella rava*, which exhibits glycolytic activity, ferments to produce acetate and succinate, thereby markedly enhancing the gut microbiota composition and fecal metabolite levels in mice [36]. Pygmaiobacter has also been identified as an inflammation-reducing agent, credited with boosting SCFA production and curbing Th17 cell polarization [37]. The increased abundance of Barnesiellaceae, Alloprevotella, and Pygmaiobacter in the RES group underscores the health-promoting impact of resveratrol supplementation in animals. *Fibrobacter succinogenes* collaborates with other key microbial players, such as *Ruminococcus flavefaciens* and *Prevotella ruminicola*, to efficiently decompose cellulose, yielding H_2_ and CO_2_ in the process. These byproducts serve as essential substrates for methanogenic archaea, thereby significantly fueling the production of methane [38]. The increased abundance of *Prevotella* may inhibit methanogens via hydrogen competition [39], which explained the higher *Prevotella* abundance in the RES group well. The notable abundance of *Fibrobacter succinogenes* in the CON group suggests that this particular group appears to be experiencing suboptimal feed utilization efficiency. The *p1088 a5 gut group*, a member of the family Pirellulaceae, is present in both the rumen and feces of ruminants, yet it displays contrasting functions. In the rumen, it is positively correlated with NH_3_-N utilization efficiency, whereas in feces, it is negatively correlated with growth performance [40]. Certain bacteria within the Lactobacillales and Bacilli, such as Lactobacillus, can produce bile salt hydrolase. This enzyme breaks down bile salts, thereby diminishing the efficiency of fat digestion and absorption [41]. In line with these observations, this study found that *p_1088_a5_gut_group*, *Bacilli*, and *Lactobacillales* were more abundant in sheep with lower growth performance. *Streptococcus equinus*, a member of the Streptococcaceae family, as well as *Treponema succinifaciens*, typically resides as a commensal in the intestines of animals. However, they can induce a range of conditions associated with indigestion and metabolic disturbances [42]. The Streptococcus bovis/Streptococcus equinus complex (SBSEC) is one of the most antibiotic-resistant groups among streptococci, posing a significant health risk to ruminants [43]. The investigation revealed that the abundance of these bacteria was significantly higher in the CON group, which implies that sheep not receiving resveratrol supplementation exhibited inferior health conditions and low feed digestion. Prior research has indicated that the relative abundance of *F082* is negatively correlated with propionate and ammonia nitrogen levels [44]. This correlation can impact the utilization rate of feed energy and protein metabolism, ultimately resulting in diminished feed efficiency. Additionally, an increase in the relative abundance of *F082* has been linked to a heightened risk of ruminal acidosis. Similarly, an increase in the abundance of *dgA 11 gut group* typically signals decreased feed efficiency and potential health concerns [45]. Our current study revealed that the CON group exhibited higher relative abundances of both *F082* and *dgA 11 gut group*, findings that align with the group’s poorer feed efficiency and overall health status.

The glycan biosynthesis and metabolism pathway encompasses the synthesis, modification, degradation, and transport of glycans. It is integral to a myriad of biological processes, such as cell recognition, signal transduction, cell-cell interactions, and immune responses. However, the over-enrichment of this metabolic pathway can disrupt the composition and function of rumen microbiota. This disruption may lead to a relative decline in beneficial microbes and an increase in pathogenic or harmful microbes, thereby disturbing the metabolic balance [46]. In this study, the pathway was found to be significantly enriched in the CON group, which might account for its relatively lower nutrient metabolic efficiency and immune capacity compared to the RES group. Additionally, enhanced glycan metabolism may promote the fermentation of recalcitrant carbohydrates, indirectly increasing ammonia nitrogen generation [47], which explained the higher concentration of NH_3_-N in the CON group well. These findings suggest that dietary supplementation with resveratrol effectively increased the abundances of beneficial gut bacteria, including *Prevotella*, *Alloprevotella*, and *Pygmaiobacter*, while concurrently reducing the abundances of potentially harmful bacteria, such as *Bacteroides*, *Alistipes*, and members of the *NK4A214 group*.

It is important to note that while this research indicates that dietary supplementation with resveratrol can lower fecal ammonia nitrogen levels, an environmentally significant finding that can mitigate the adverse environmental effects of animal husbandry. Numerous studies have demonstrated that dietary resveratrol supplementation can enhance feed efficiency and nitrogen digestibility in ruminants [2,4]. Nevertheless, since the study lacks direct measurements of feed efficiency or nitrogen retention rate, the current findings can only suggest an improvement in the animals’ nitrogen utilization efficiency indirectly. Typically, ammonia nitrogen arises when ingested proteins are not fully absorbed and utilized by animals; instead, they are excreted as urea and subsequently broken down by gut microbes. Thus, the observed decrease in ammonia nitrogen content due to resveratrol may indicate more efficient protein absorption and utilization by the animals, potentially boosting feed efficiency. Nevertheless, this hypothesis requires further substantiation through the direct assessments of feed efficiency and nitrogen retention rate.

## 5. Conclusions

Taken together, dietary supplementation with resveratrol altered the fecal fermentation characteristics by increasing the fecal pH value while reducing the concentration of NH_3_-N, but had no effect on the concentration or proportion of VFA. The addition of resveratrol decreased the richness, Shannon index, and inverse Simpson index of fecal microbiota. The relative abundances of Planctomycetota, *Bacteroides*, *Alistipes*, and *NK4A214 group* were higher in the CON group than in the RES group. Additionally, the relative abundance of the glycan biosynthesis and metabolism pathway was also higher in the CON group. ANOSIM revealed significant differences between the CON and RES groups, and LEfSe analysis identified 27 microbial biomarkers. This study indicates that dietary supplementation with resveratrol can alter fecal fermentation characteristics, microbial diversity, and community composition. Dietary 100 mg resveratrol/kg feed supplementation could serve as an environmentally friendly additive to reduce fecal ammonia nitrogen emissions in ruminants. However, it should be admitted that the trial period only covered the growing phase; the long-term effects of continuous resveratrol supplementation on reproductive performance and sustained fecal pollution mitigation require further investigation. The findings of this research can offer a post-digestion perspective for evaluating the application of resveratrol in ruminant production.

## Figures and Tables

**Table 1:** Feed ingredients and their chemical composition in the basal diet.

Ingredient	Proportion, g/kg	Nutritional Component	Value
Corn	247.5	Metabolizable energy, MJ/kg	10.06
Soybean meal	110.0	Crude protein, %	13.23
Wheat bran	125.0	Neutral detergent fiber, %	40.17
Wheat straw	287.5	Acid detergent fiber, %	24.58
Peanut straw	200.0	Ether extract, %	2.61
Calcium hydrogen phosphate	10.0	Calcium, %	0.87
Limestone	5.0	Phosphorus, %	0.50
Salt	5.0		
Premix ^(1)^	10.0		

^(1)^ Premix provided the following per kg of DM: 1400 mg of Fe, 1200 mg of Zn, 250 mg of Cu, 900 mg of Mn, 100,000 IU of vitamin A, 27,000 IU of vitamin D3, and 800 IU of vitamin E.

**Table 2:** Effect of resveratrol supplementation on fecal fermentation characteristics of Hu sheep.

Item	CON	RES	SEM	*p*-Value
pH value	7.26	7.41	0.041	0.017
Ammonia nitrogen, mg/dL	24.51	21.91	0.659	0.013
Concentration, mmol/L				
Acetate	48.64	45.98	1.279	0.210
Propionate	11.61	10.52	0.461	0.112
Isobutyrate	0.46	0.49	0.029	0.416
Butyrate	4.50	4.42	0.198	0.790
Isovalerate	0.56	0.56	0.057	0.994
Valerate	0.61	0.57	0.041	0.501
Branched-chain volatile fatty acids	1.62	1.62	0.120	0.912
Total volatile fatty acids	66.37	62.53	1.919	0.209
Proportion, %				
Acetate	73.34	73.60	0.550	0.746
Propionate	17.44	16.80	0.336	0.194
Acetate/propionate	4.22	4.40	0.118	0.298
Isobutyrate	0.68	0.79	0.047	0.123
Butyrate	6.78	7.00	0.163	0.369
Isovalerate	0.83	0.90	0.082	0.588
Valerate	0.91	0.90	0.052	0.903
Branched-chain volatile fatty acids	2.43	2.59	0.167	0.579

CON, the control group which was given a basal diet; RES, the treatment group which was fed the same basal diet supplemented with resveratrol at 100 mg/kg of feed.

**Table 3:** Effect of resveratrol supplementation on the fecal bacterial alpha-diversity of Hu sheep.

Item	CON	RES	SEM	*p*-Value
Richness	1326.20	1083.00	72.887	0.049
Chao1	1459.60	1221.56	77.359	0.066
ACE	1432.59	1194.75	76.643	0.066
Shannon index	6.28	5.79	0.106	0.013
Simpson index	0.9951	0.9886	0.002	0.056
Inverse Simpson index	219.70	113.92	24.922	0.017

CON, the control group which was given a basal diet; RES, the treatment group which was fed the same basal diet supplemented with resveratrol at 100 mg/kg of feed.

**Table 4:** Effect of resveratrol supplementation on fecal bacterial community composition at the phylum level (>0.1%) of Hu sheep.

Phylum Name	CON	RES	SEM	*p*-Value
Firmicutes	58.71	58.89	1.459	0.931
Bacteroidota	29.57	32.70	1.255	0.119
Spirochaetota	4.66	3.69	0.981	0.512
Actinobacteriota	2.40	1.66	1.159	0.841
Verrucomicrobiota	2.45	0.61	0.621	0.151
Fibrobacterota	0.57	1.70	0.637	1.000
Planctomycetota	1.15	0.06	0.192	0.032
Proteobacteria	0.30	0.29	0.143	0.974
Campilobacterota	0.03	0.21	0.073	0.841

CON, the control group which was given a basal diet; RES, the treatment group which was fed the same basal diet supplemented with resveratrol at 100 mg/kg of feed.

**Table 5:** Effect of resveratrol supplementation on fecal bacterial community composition at the genus level (>1.0%) of Hu sheep.

Genus Name	CON	RES	SEM	*p*-Value
*Christensenellaceae R-7 group*	14.67	11.02	1.836	0.210
*Rikenellaceae RC9 gut group*	7.07	11.05	1.357	0.085
*UCG-005*	8.38	8.39	1.063	0.996
*Bacteroides*	10.50	5.39	0.741	0.002
*RF39*	7.14	3.13	1.530	0.095
*Treponema*	4.66	3.69	0.981	0.512
*Alistipes*	4.19	3.10	0.264	0.020
*UCG-010*	1.76	4.19	0.955	0.310
*Ruminococcus*	2.01	3.58	0.592	0.421
*Clostridia UCG-014*	3.51	2.03	0.585	0.113
*Muribaculaceae*	1.71	3.71	1.698	0.421
*Lachnospiraceae AC2044 group*	1.34	2.68	0.784	1.000
*UCG-002*	1.85	2.05	0.438	0.775
*Prevotella*	0.49	2.86	0.546	0.022
*Bacteroidales RF16 group*	0.66	2.46	0.954	0.222
*Monoglobus*	1.20	1.87	0.343	0.207
*Eubacterium coprostanoligenes group*	2.16	0.90	0.539	0.151
*Lachnospiraceae NK4A136 group*	1.25	1.78	0.252	0.180
*Akkermansia*	2.36	0.59	0.623	0.151
*Eubacterium siraeum group*	1.13	1.73	0.405	0.337
*Anaerostipes*	1.16	1.66	0.685	0.548
*NK4A214 group*	1.84	0.62	0.250	0.011
*Bifidobacterium*	1.60	0.85	0.907	0.421
*Fibrobacter*	0.57	1.70	0.637	1.000

CON, the control group which was given a basal diet; RES, the treatment group which was fed the same basal diet supplemented with resveratrol at 100 mg/kg of feed.

**Table 6:** Effect of resveratrol supplementation on the relative abundance of the predicted metabolic pathways in the fecal bacterial community.

Metabolic Pathway	CON	RES	SEM	*p*-Value
Protein families: genetic information processing	18.35	18.29	0.079	0.565
Protein families: signaling and cellular processes	11.75	11.82	0.086	0.575
Carbohydrate metabolism	8.93	8.85	0.061	0.381
Amino acid metabolism	6.70	6.78	0.039	0.141
Protein families: metabolism	6.07	6.10	0.025	0.426
Metabolism of cofactors and vitamins	3.91	3.98	0.042	0.307
Energy metabolism	3.83	3.84	0.027	0.775
Translation	3.58	3.55	0.029	0.382
Replication and repair	3.04	3.00	0.027	0.282
Nucleotide metabolism	2.65	2.64	0.017	0.764
Unclassified: metabolism	2.50	2.48	0.017	0.402
Membrane transport	2.34	2.41	0.047	0.295
Poorly characterized	2.15	2.17	0.010	0.384
Signal transduction	2.07	2.02	0.024	0.238
Glycan biosynthesis and metabolism	1.98	1.89	0.020	0.011
Cellular community—prokaryotes	1.85	1.85	0.016	0.934
Lipid metabolism	1.77	1.79	0.018	0.554
Biosynthesis of other secondary metabolites	1.56	1.58	0.014	0.400
Unclassified: signaling and cellular processes	1.50	1.55	0.027	0.284
sorting and degradation	1.48	1.47	0.005	0.295
Folding	1.48	1.47	0.005	0.309
Metabolism of other amino acids	1.11	1.11	0.007	0.841
Cell motility	1.07	1.02	0.050	0.477

CON, the control group which was given a basal diet; RES, the treatment group which was fed the same basal diet supplemented with resveratrol at 100 mg/kg of feed.

## Data Availability

The raw sequencing data in this study were deposited in the NCBI Sequence Read Archive (SRA) database, and the assigned accession number is PRJNA1274161.

## Abbreviations

The following abbreviations are used in this manuscript:

PCoA	Principal co-ordinate analysis
ANOSIM	Analysis of similarities
LEfSe	Linear discriminant analysis effect size
VFA	Volatile fatty acids
SRA	Sequence read archive
ASVs	Amplicon sequence variants
LDA	Linear discriminant analysis
PICRUSt	Phylogenetic investigation of communities by reconstruction of unobserved states
SCFA	Short-chain fatty acids
